# Wheat with greatly reduced accumulation of free asparagine in the grain, produced by CRISPR/Cas9 editing of asparagine synthetase gene *TaASN2*

**DOI:** 10.1111/pbi.13573

**Published:** 2021-03-28

**Authors:** Sarah Raffan, Caroline Sparks, Alison Huttly, Lucy Hyde, Damiano Martignago, Andrew Mead, Steven J. Hanley, Paul A. Wilkinson, Gary Barker, Keith J. Edwards, Tanya Y. Curtis, Sarah Usher, Ondrej Kosik, Nigel G. Halford

**Affiliations:** ^1^ Department of Plant Sciences Rothamsted Research Harpenden UK; ^2^ Department of Computational and Analytical Sciences Rothamsted Research Harpenden UK; ^3^ Functional Genomics School of Biological Sciences University of Bristol Bristol UK; ^4^ Curtis Analytics Limited Rothamsted Research Campus Harpenden UK; ^5^ Present address: School of Biological Sciences Life Sciences Building University of Bristol 24 Tyndall Avenue Bristol BS8 1TQ UK; ^6^ Present address: Department of Biosciences University of Milan Via Celoria 26 Milano 20133 Italy

**Keywords:** asparagine synthetase, acrylamide, food safety, wheat, grain composition, genome editing, CRISPR/Cas9, asparagine, amino acids

## Abstract

Free asparagine is the precursor for acrylamide, which forms during the baking, toasting and high‐temperature processing of foods made from wheat. In this study, CRISPR/Cas9 was used to knock out the asparagine synthetase gene, *TaASN2,* of wheat (*Triticum aestivum*) cv. Cadenza. A 4‐gRNA polycistronic gene was introduced into wheat embryos by particle bombardment and plants were regenerated. T1 plants derived from 11 of 14 T0 plants were shown to carry edits. Most edits were deletions (up to 173 base pairs), but there were also some single base pair insertions and substitutions. Editing continued beyond the T1 generation. Free asparagine concentrations in the grain of plants carrying edits in all six *TaASN2* alleles (both alleles in each genome) were substantially reduced compared with wildtype, with one plant showing a more than 90 % reduction in the T2 seeds. A plant containing edits only in the A genome alleles showed a smaller reduction in free asparagine concentration in the grain, but the concentration was still lower than in wildtype. Free asparagine concentration in the edited plants was also reduced as a proportion of the free amino acid pool. Free asparagine concentration in the T3 seeds remained substantially lower in the edited lines than wildtype, although it was higher than in the T2 seeds, possibly due to stress. In contrast, the concentrations of free glutamine, glutamate and aspartate were all higher in the edited lines than wildtype. Low asparagine seeds showed poor germination but this could be overcome by exogenous application of asparagine.

## Introduction

The accumulation of free (soluble, non‐protein) asparagine in grains, tubers, storage roots, beans and other crop products is the focus of much research because free asparagine is the precursor for acrylamide (CH_2_ = CHC(O)NH_2_) formation during cooking and processing (Mottram *et al.,* 2002; Stadler *et al.,* 2002). Acrylamide is a processing contaminant that forms from free asparagine and reducing sugars within the Maillard reaction (Mottram *et al.,* 2002; Stadler *et al.,* 2002; Zyzak *et al.,* 2003). It was discovered in food in 2002 (Tareke *et al.,* 2002) and is associated with fried, baked, roasted and toasted foods, including bread, biscuits, cakes, pies, batter and breakfast cereals (reviewed by Raffan and Halford, 2019).

Acrylamide is classed as a Group 2a carcinogen by the International Agency for Research on Cancer (IARC) (1994). In 2006, a United Nations’ Food and Agriculture Organisation and World Health Organisation Joint Expert Committee on Food Additives (JECFA) report stated that the potential cancer‐causing effects of acrylamide in the diet were a concern (JECFA, 2006), and the European Food Safety Authority (EFSA) Panel on Contaminants in the Food Chain (CONTAM) came to a similar conclusion in 2015 (CONTAM Panel, 2015). This led to the adoption of European Commission (EC) Regulation (EU) 2017/2158 (European Commission, 2017), which states that ‘acrylamide in food potentially increases the risk of developing cancer for consumers in all age groups’, sets Benchmark Levels for acrylamide in different food types, and threatens the introduction of Maximum Levels (levels above which it would be illegal to sell a food product). It also sets out compulsory risk management measures for food businesses to follow. Subsequently (November 2019), the European Commission issued Recommendation (EU) 2019/1888, which stated that levels of acrylamide should be monitored in a range of foods that did not fall within the scope of Regulation (EU) 2017/2158, including a number of bakery and cereal products (European Commission, 2019).

Methods for reducing acrylamide in food products have been compiled in an ‘Acrylamide Toolbox’ by FoodDrinkEurope (2019). Some have been successful, but they are not applicable to all food types, are often expensive to implement and may have detrimental effects on product quality. The food industry would therefore benefit from the availability of raw materials with lower acrylamide‐forming potential, and the determining factor for acrylamide formation in products made from wheat (*Triticum aestivum*) and rye (*Secale cereale*) grains, and probably those of other cereals, is the concentration of free asparagine (reviewed by Raffan and Halford, 2019). In wheat, for example, Halford *et al*. (2007) reported *R* ^2^ values of 0.998 and 0.956 for the relationship between free asparagine concentration and acrylamide formation in heated flour produced from grain of field‐ and pot‐grown wheat, respectively. Similarly, Curtis *et al*. (2009) reported an *R* ^2^ value of 0.9945 for a selection of field‐ and pot‐grown samples with a wide range of free asparagine concentrations (0.67 to 62 mmol per kg, with the higher concentrations in grain from sulphur‐deprived plants).

The accumulation of free asparagine in wheat grain is responsive to environmental and crop management factors, increasing, for example, in response to sulphur deficiency and pathogen infection (reviewed by Raffan and Halford, 2019). Different varieties also show a wide range of free asparagine concentrations even under optimal conditions (Curtis *et al.,* 2018b). A network has been constructed to describe the factors that contribute to the genetic control of asparagine metabolism (Curtis *et al.,* 2018a), and at the heart of that network are genes encoding asparagine synthetases; enzymes that catalyse the ATP‐dependent transfer of an amino group from glutamine to aspartate to form glutamate and asparagine (Gaufichon *et al.,* 2010). Wheat has five asparagine synthetase genes per genome: *TaASN1*, *TaASN2*, *TaASN3.1*, *TaASN3.2* and *TaASN4*, although some varieties lack a *TaASN2* gene on the B genome (Raffan and Halford, 2021; Xu *et al.,* 2018). *TaASN2* is expressed seed‐specifically, with highest expression in the embryo (Curtis *et al.,* 2019; Gao *et al.,* 2016). The other three genes are more widely expressed in the plant, and *TaASN1* is responsive to salt stress, osmotic stress and abscisic acid (Wang *et al.,* 2005).

The aim of this study was to target the *TaASN2* gene in wheat cv. Cadenza using CRISPR/Cas9, to reduce asparagine synthetase gene expression in the grain without affecting its expression elsewhere in the plant. Our hypothesis was that the relatively low expression of the other asparagine synthetase genes in the grain (Curtis *et al.,* 2019; Gao *et al.,* 2016) would provide sufficient asparagine for protein synthesis but not allow free asparagine to accumulate to the levels seen when a functional *TaASN2* gene is present.

## Results

### Designing gRNA sequences for the CRISPR/Cas9 system

**Figure 1:**
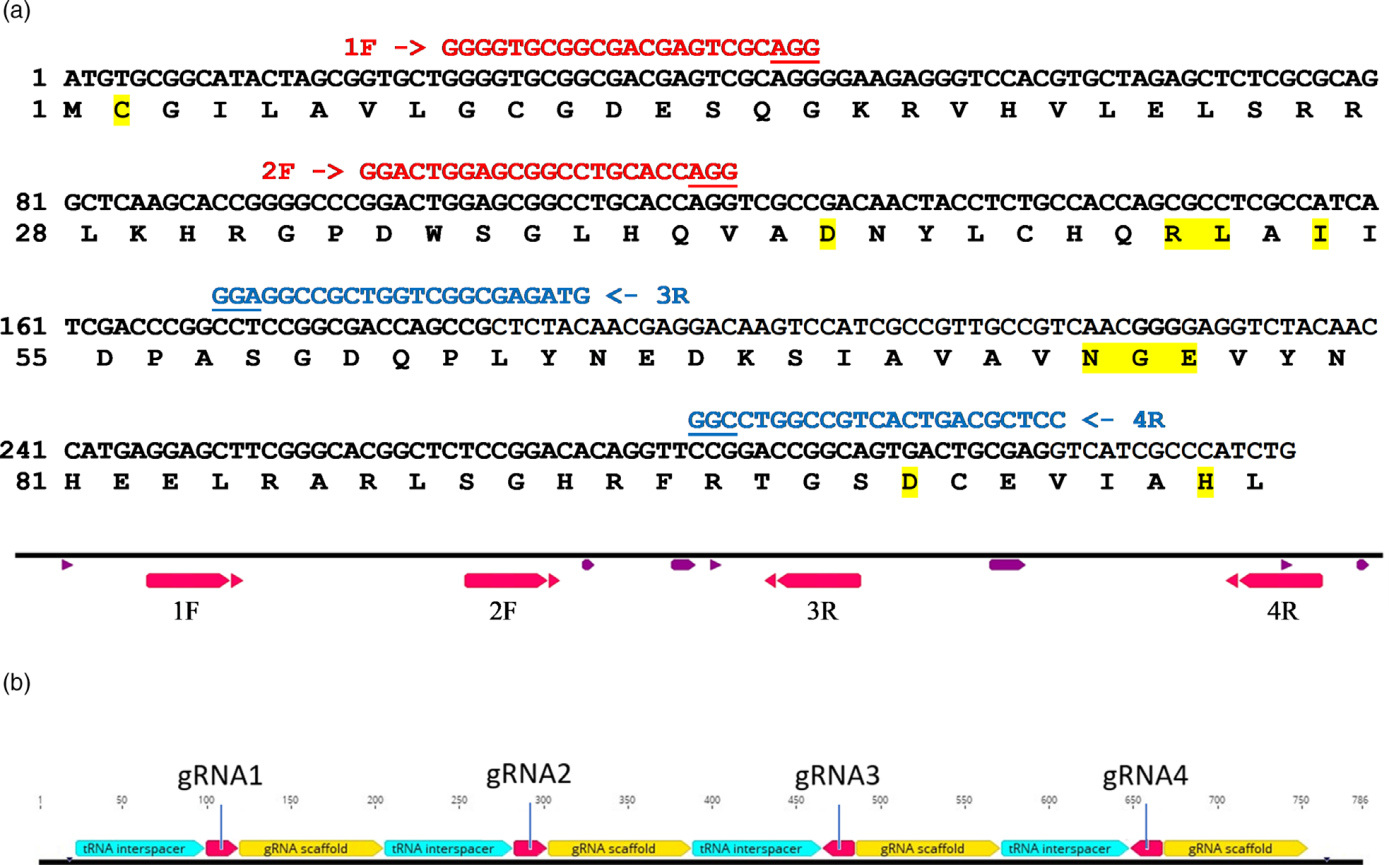
(a) Top: Nucleotide and derived amino acid sequence of the target region in the first exon of *TaASN2* showing the binding positions of the gRNAs. The forward gRNAs are shown in red and the reverse in blue, with PAM sequences underlined. Residues known to be important in glutamine binding (Gaufichon *et al.,* 2010) are highlighted in yellow. The nucleotide sequence shown is that of the A genome *TaASN2* gene, but the nucleotide sequences of the B and D genome *TaASN2* genes are identical at the gRNA binding sites. Bottom: schematic diagram showing the relative positions of the gRNA binding sites and the nucleotide sequences encoding key residues (purple). (b) Diagrammatic representation of the polycistronic gene comprising the four gRNAs separated by tRNA sequences.

Nucleotide sequences of the asparagine synthetase genes of wheat (*Triticum aestivum*) cv. Cadenza were used to design guide RNAs (gRNAs) to target the first exon of all three homeologues of the *TaASN2* gene but not the other asparagine synthetase genes (*TaASN1*, *TaASN3.1*, *TaASN3.2* and *TaASN4*). The gRNAs all showed at least two mismatches to the other *TaASN* genes in the PAM‐proximal region shown to be crucial for Cas9 function (Kuscu *et al.,* 2014), with the gRNA4 position also lacking the requisite PAM sequence. The first exon of *TaASN2* encodes the glutamine amidotransferase domain of the protein (Gaufichon *et al.,* 2010; Xu *et al.,* 2018). EnsemblPlants database references for the genes are given in Data S1 (Sheet 2). The targeting sequences were attached to an optimized gRNA scaffold structure (Dang *et al.,* 2015) to enable the gRNAs to interact with the Cas9 nuclease. The gRNAs, selected using Cas9 gRNA design tools, RGEN (Park *et al.,* 2015) and DESKGEN (Hough *et al.,* 2016), were: 1F (GGGGTGCGGCGACGAGTCGCAGG), 2F (GGACTGGAGCGGCCTGCACCAGG), 3R (GTAGAGCGGCTGGTCGCCGGAGG) and 4R (CCTCGCAGTCACTGCCGGTCCGG), with F and R denoting forward or reverse orientations and the PAM (which is not present in the final gRNAs) underlined. Their binding sites are shown in Figure 1a, along with the amino acid sequence encoded by the target region of the gene, with residues important for the activity of the enzyme highlighted.

The four gRNA‐encoding DNAs were incorporated into a single, polycistronic gene, separated by tRNA sequences (Figure 1b), based on the system developed by Xie *et al*. (2015). This system exploits the endogenous tRNA‐processing system to generate multiple gRNAs from a single transgene, enabling multiplex editing. The construct was assembled using a Golden Gate system (Engler *et al.,* 2008) in plasmid pRRes209.481 (Alison Huttly, Rothamsted Research) to make pRRes209.481.ASN2. The successful generation of the plasmid was confirmed by nucleotide sequence analysis.

### Wheat transformation

Plasmid pRRes209.481.ASN2 was introduced into wheat cv. Cadenza embryos by particle bombardment, together with pRRes.486, which carries a *Cas9* gene codon‐optimized for wheat, driven by a maize ubiquitin promoter, and a plasmid, pRRes1.111 (Alison Huttly, Rothamsted Research), containing a selectable marker gene (*bar*), which imparts tolerance to glufosinate ammonium. Transformed embryos were recovered, cultured on selective medium and grown on to produce plants. Six separate bombardments were performed, resulting in 92 transformed plants, 77 (84%) of which showed *Cas9* integration. The T0 plants were self‐pollinated to generate T1 seed.

### Polymerase chain reaction and next generation sequencing analysis of T1 plants

Seedlings were grown from the T1 seed of 14 of the T0 plants. These 14 T0 plants were selected solely on the basis that they were growing well. DNA from 167 potentially edited T1 plants, comprising between 9 and 20 plants grown from seed of each of the 14 selected T0 plants, were then analysed by next generation sequencing (NGS). Editing events were detected in 107 (64%) of these plants, derived from 11 of the 14 T0 plants. Of these, 55 (33% of the total plants; 51% of the edited plants) were edited in all three genomes, at least heterozygously, and these were derived from 9 of the 14 selected T0 plants. Edits were not detected in 37 plants (22%) and the remaining 21 plants showed an insufficient read number to be analysed.

Six T1 plants, 23, 41, 59, 99, 126 and 178 were selected for further study on the basis of the NGS analysis. The editing events detected in these plants are shown in Table 1. Plants 23 and 59 were total nulls; that is with edits in both alleles of each genome. Plant 99 was edited in both alleles of the A and D genomes, but its B genome alleles were tentatively assigned to wildtype (the NGS coverage of those alleles was poor). Plants 41 and 178 were A genome nulls; that is both alleles of the A genome gene were edited but the B and D genomes genes were unaffected. These A genome nulls were of interest because the A genome *TaASN2* gene had been shown to be relatively highly expressed (Curtis *et al.,* 2019). Line 126 was an unedited control.

**Table 1:** Editing events in the *TaASN2* alleles and homeoalleles of the T1 generation of selected lines of wheat (*Triticum aestivum*) cv. Cadenza after editing with CRISPR/Cas9. The edits at the four gRNA positions are shown for every genotype. Insertion and deletion events are represented by + and – signs, respectively, together with the number of additional or missing nucleotides. G → T indicates a guanine to thymine substitution. WT = wildtype

Line	Status	Genome	Biallelic/Homozygous	Allele	gRNA position			
					1F	2F	3R	4R
23	Triple null	A	Biallelic	1			+1	+1
				2			G → T	+1
		B	Biallelic	1				+1
				2			+1	+1
		D	Biallelic	1			+1	−18
				2				−2
30	Triple null	A	Homozygous	1 & 2			+1	−1
		B	Homozygous	1 & 2			+1	−1
		D	Homozygous	1 & 2				−1
59	Triple null	A	Biallelic	1	−11	+1	−2	ND
				2	+1	−4	+1	
		B	Biallelic	1			G → T	ND
				2				+1
		D	Homozygous	1 & 2	−4	−11	+1	
99	A and D genome null	A	Biallelic	1				+1
				2			−1	−2
		B	Homozygous	1&2	WT^†^			
		D	Biallelic	1				+1
				2			+1	−1
41	A genome null	A	Homozygous	1 & 2	−14		+1	ND
		B	Homozygous	1 & 2	WT			
		D	Homozygous	1 & 2	WT			
178	A genome null	A	Homozygous	1 & 2			+1	
		B	Homozygous	1 & 2	WT			
		D	Homozygous	1 & 2	WT			

ND, not determined due to ambiguous data close to the end of the reads.

^†^ Provisional assignment based on poor coverage in the NGS data.

### NGS analysis of editing in T2 plants

**Figure 2:**
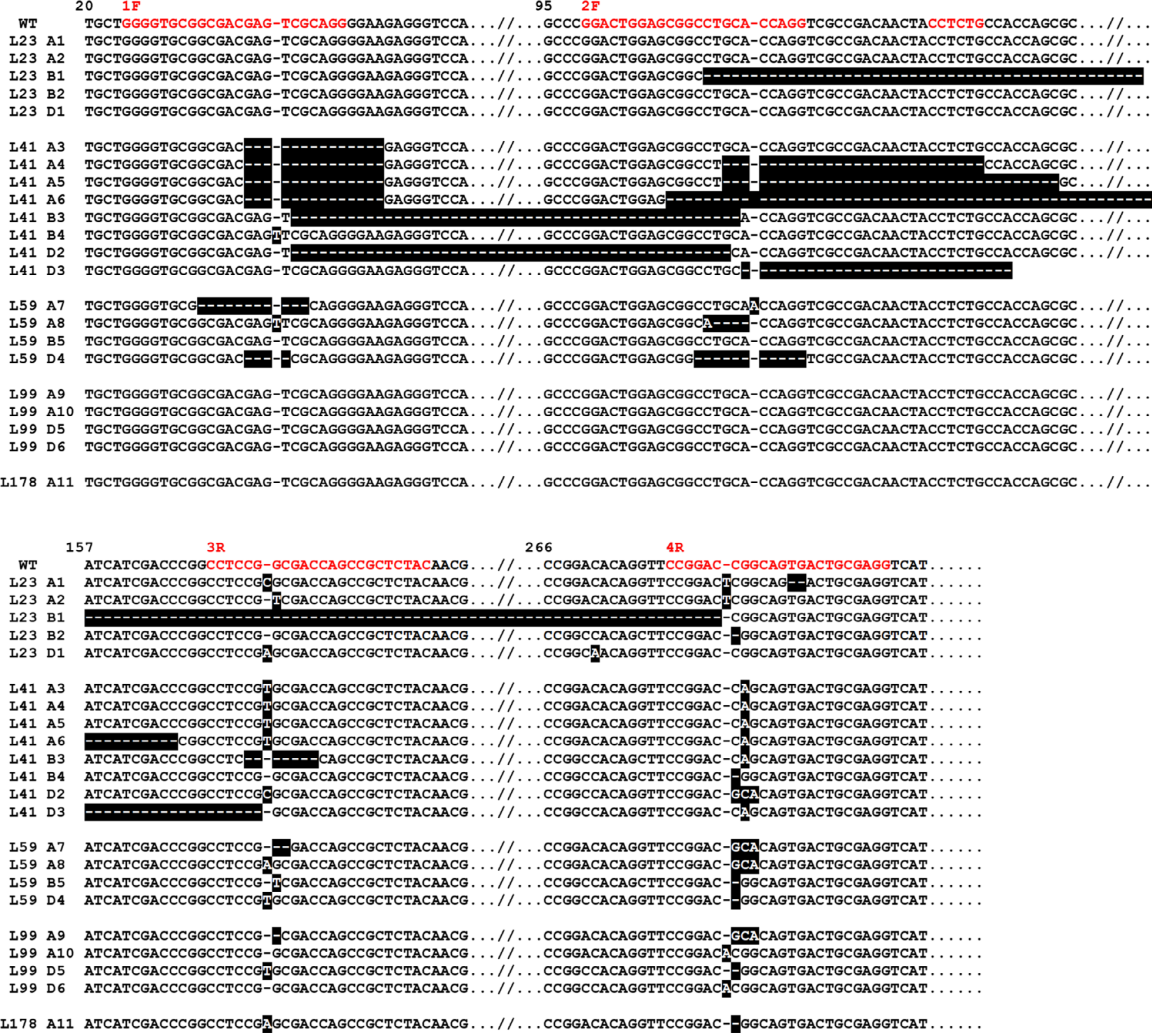
Nucleotide sequences showing the edited *TaASN2* alleles present in the T2 generation of wheat (*Triticum aestivum*) cv. Cadenza plants after editing with CRISPR/Cas9. The plants were from lines 23, 41, 59, 99 and 178, as indicated. The A genome wildtype sequence is shown at the top, with the gRNA binding sites in red. Edits are highlighted in black. SNPs present in the wildtype sequences of the genes from genomes B and D are not highlighted. The different alleles are numbered, A1 to A11, B1 to B5 and D1 to D6.

T2 plants were grown from the seeds of plants 23, 59, 99, 41 and 98, with plants derived from plant 23 referred to as plants of line 23, and so on. These plants were also analysed by NGS and the different alleles observed in individual plants are shown in Figure 2. The alleles were still segregating in some cases, resulting in different plants within each line having different combinations of alleles. The NGS data on these plants gave good coverage for all three genomes.

Plants of line 23 (numbered 8, 12, 18, 33, 39, 45, 55, 60, 75 and 77) showed the same two A genome alleles (labelled A1 and A2 in Figure 2) as seen in the T1 generation, both of which encode truncated proteins (Table 1). Plants 12 and 60 were homozygous for allele A1, with the rest biallelic. Two edited B genome alleles (B1 and B2 in Figure 2) were also present, one showing a deletion of 173 bases between the 2F and 4R positions and the second a deletion of a single base at 4R. Both edits cause frameshifts that bring stop codons into frame, resulting in the genes encoding truncated proteins. Plants 12 and 60 were homozygous for allele B1 while the other plants were biallelic. Neither of these alleles was observed in the T1 generation, indicating that editing had continued into the T2 generation, resulting in novel edited alleles appearing.

A single, novel, edited D genome allele was also present (D1 in Figure 2), also encoding a truncated protein. As with the B genome alleles, this indicates that editing was continuing into the T2 generation. Plants 33, 45, 60 and 75 were homozygous for this allele. A wildtype allele also appeared in this generation, with plants 8, 12 and 55 homozygous for this allele and the others showing a mix of wildtype and edited reads.

Ten plants were analysed for line 41 (10, 15, 21, 29, 38, 43, 46, 61, 69 and 82). One main allele was recorded in the A genome (A3 in Figure 2), showing a deletion of 14 bases at position 1F and the addition of a single thymine at 3R, causing a frameshift, plus a guanine to adenine substitution at 4R. Plants 15, 21, 29, 43, 46, 61 and 69 were homozygous for this allele. Plant 10 showed a second allele (A4 in Figure 2) with a second deletion of 27 bases at 2F, while plant 82 showed another variant (A5) with a longer second deletion of 35 bases at 2F, and plant 38 a fourth variant (A6) with a deletion of 59 bases between the 2F and 3R positions.

The presence of these four different A genome alleles is strong evidence of continued editing. Furthermore, while the 14 base pair deletion at 1F was present in the single A genome allele seen in the T1 generation (Table 1), the longer deletions at 2F were not, suggesting that these deletions were later edits added to the allele seen in the T1 generation. In addition, T1 plant 41 was originally shown to be an A genome null, but by the T2 generation two edited alleles had appeared in each of the B and D genomes (B3, B4, D2 and D3 in Figure 2). Three T2 plants (10, 43 and 61) were homozygous for allele B3 and two (29 and 69) for B4, while the remaining plants were biallelic with both edited alleles. Plants 38, 43, 46 and 61 were homozygous for D2, while 10 and 21 were homozygous for D3, with the other plants biallelic with both alleles.

Eleven plants (2, 6, 16, 26, 37, 54, 59, 67, 80, 84 and 89) were analysed for line 59. The two edited A genome alleles seen in the T1 generation (Table 1) were present in the T2 generation, with no indication of further editing (A7 and A8 in Figure 2). Plants 16, 26 and 37 were homozygous for A7, while the remaining plants were biallelic, carrying both edited alleles. A7 encodes a protein of 577 amino acid residues, only four shorter than the wildtype, but residues 11 to 56 are changed due to the coding sequence going out of frame and then back in again. This region includes key residues required for glutamine binding (Figure 1a). Allele A8 encodes a protein of only 80 amino acid residues.

The plants in line 59 were all homozygous for a single edited B genome allele (B5 in Figure 2). This allele, which has a frameshift at the 96^th^ codon, is very similar to one of the alleles in the T1 generation (Table 1), except that editing at the 4R position in the T1 generation was unclear from the NGS data. The plants were also homozygous for the single edited D genome allele seen in the T1 generation (Table 1), labelled as D4 in Figure 2. This allele has a frameshift in the 13^th^ codon and encodes a truncated protein of only 76 residues.

Nine plants were analysed for line 99 (20, 24, 28, 50, 52, 58, 63, 68 and 72). There were two different edited A genome alleles present (A9 and A10 in Figure 2). A9 showed the loss of one base at the 3R position. This was also seen in one of the T1 alleles (Table 1) but that allele also showed a deletion of two bases at the 4R position, whereas A9 did not but did have three changed bases (CGG to GCA) at that position. A10 was identical to one of the edited alleles seen in the T1 generation (Table 1), with the addition of a single adenine at position 4R. Plants 20 and 52 were biallelic for A9 and A10, while plants 28, 50 and 58 were homozygous for A9 and plants 63, 68 and 72 were homozygous for A10. Plant 24 was heterozygous with allele A9 and a wildtype allele, something that had not been seen in the T1 generation.

The wildtype B genome alleles provisionally identified in the T1 generation were confirmed in the T2 generation. In the D genome, the two alleles identified in the T1 generation (Table 1) were shown to be present again (D5 and D6 in Figure 2). These alleles encode truncated proteins of 81 and 97 amino acid residues, respectively. Plants 24 and 72 were biallelic with both alleles, D5 and D6, while plants 50, 58 and 68 were all homozygous for D5, and plants 28, 52 and 63 were homozygous for D6. As with the A genome, a wildtype D genome allele reappeared in this generation, with plant 20 heterozygous for D5 and wildtype.

Lastly, ten plants (3, 13, 23, 35, 42, 51, 64, 66, 76 and 86) were analysed for the other A genome null from the T1 generation, line 178. Unlike line 41, no additional editing was observed, with all plants homozygous for the edited A genome allele seen in the T1 generation (Table 1), and wildtype for the B and D genome alleles. The A genome edited allele (A11 in Figure 2) has a single additional adenine at the 3R position, causing a frame shift in the sequence and the introduction of a stop codon at the 82^nd^ codon.

### Free amino acid concentrations in the grain

The concentrations of free amino acids in the grain harvested from the T1 plants (i.e. the T2 grain) were measured by GC‐MS. Five single seeds were analysed for each plant due to the limited amount of seed available, and the complete dataset is given in Data S3 (Sheet 4).

The wildtype control seeds showed free asparagine concentrations ranging from 1.1 to 2.3 mmol/kg (rounding to two significant figures) with an average value of 1.6 mmol/kg, and the seeds of plant 126 (unedited control) showed a similar range from 0.88 to 2.8 mmol/kg, with an average of 1.5 mmol/kg. The relatively wide range in concentrations was explained by the fact that single seeds were being analysed. The averages were relatively low compared with the concentrations reported previously for Cadenza grown in the field (Curtis *et al.,* 2018b), but free asparagine is highly responsive to environmental factors so this was not particularly surprising. The fact that the unedited control was so similar to wildtype meant that it was not considered necessary to analyse it separately in the T3 seed.

**Figure 3:**
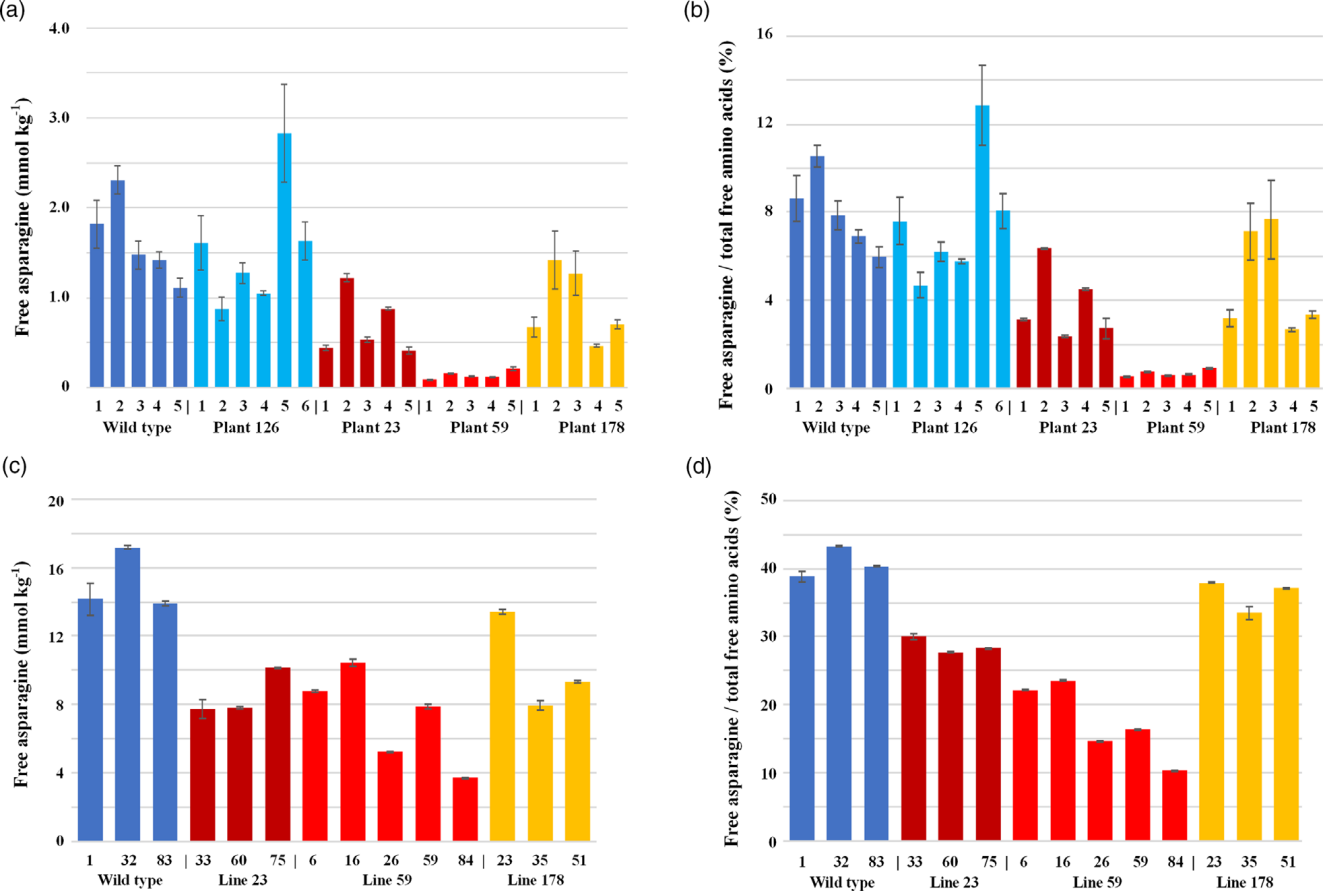
Free asparagine concentrations in the grain of edited wheat (*Triticum aestivum*) cv. Cadenza plants/lines 23, 59 and 178, and wildtype controls. (a) T2 generation, with free asparagine concentrations shown for five individual seeds from each plant. (b) As for A but with free asparagine concentration shown as a proportion (%) of the free amino acid pool. (c) T3 generation, with free asparagine concentration shown for wholemeal flour prepared from the grain of different plants within each line (i.e. the progeny of the plants shown in a and b), as indicated. (d) As for c but with free asparagine concentration shown as a proportion (%) of the free amino acid pool. The graphs show the means and standard errors from three technical reps.

Seeds of plants 99 and 41 showed no reduction in free asparagine concentration; indeed, plant 99 showed relatively high concentrations of free asparagine, particularly in two of the seeds (5.8 mmol/kg and 3.9 mmol/kg) (Data S3, Sheet 4). These results reflected the NGS data on the plants subsequently derived from T2 seeds, which showed line 99 to have wildtype B genome alleles, with editing still taking place in the plants of both lines, meaning that different seeds derived from the same plant were now carrying different edited alleles. These lines were not considered further as potential low asparagine genotypes, and we focussed on the edited lines that showed most stability, judging from the T1 and T2 generation NGS data. These were lines derived from plants 23, 59 (both triple nulls) and 178 (A genome null). The free asparagine concentrations for the T2 seeds from these plants are shown graphically in Figure, both *per se* (Figure 3a) and as a proportion of the free amino acid pool (Figure 3b).

Seeds of plant 178 had an average free asparagine concentration of 0.91 mmol/kg (56% of the wildtype average). Plant 23 showed variable levels of free asparagine but its average concentration was only 43% of the wildtype average at 0.70 mmol/kg. However, the other triple null, plant 59, was even lower than that, with a relatively narrow range of 0.10 to 0.22 mmol/kg and an average concentration of 0.14 mmol/kg, only 9% of the wildtype average.

The concentration of total free amino acids was similar across all the plants, with a mean of 20 mmol/kg in the wildtype, plant 126 and plant 59, 18 mmol/kg in plant 23, and 19 mmol/kg in plant 178. This meant that free asparagine represented a much lower proportion of the free amino acid pool in plants 23 and 59, and to a lesser extent in plant 178 (Figure 3b). The figure for plant 59 ranged from 0.55% to 0.92%, with a mean of 0.70%, compared with a mean of 8.0% for the wildtype and 7.4% for line 126. The means for plants 23 and 178 were 3.8% and 4.8%, respectively.

The T2 seed of lines 23, 41, 59, 99 and 178 were sown in pots in a glasshouse with five plants per 254 mm pot, randomized according to a statistical blocking method. A wildtype control plant was sown in each of the pots to allow for comparison between the pots. Plants of lines 23, 59 and 178 were selected for amino acid analysis based on the NGS data; this reduced the number of reps per line but ensured that, for example, all the plants of line 23 that were analysed were genuine triple nulls. The alleles (Figure 2) present in the plants that were analysed are given in Table 2, along with the predicted length in amino acid residues of the encoded proteins. In all cases, the proteins encoded by these alleles are drastically truncated compared with the wildtype and could not be functional, with the exception of the 577 amino acid protein encoded by allele A7 (Figure 2) in line 59, which lacks a glutamine binding domain. Two plants of this line were homozygous for this allele while the other three were biallelic for alleles A7 and A8 (Table 2). All five plants were homozygous for the B and D genome alleles (Table 2). Plants of line 23 also included two that were biallelic for alleles A1 and A2 and these were also biallelic in the B genome, with alleles B1 and B2. The other was homozygous for alleles A1 and B2.

**Table 2:** *TaASN2* alleles present in the T2 generation plants of lines 23, 59 and 178 from which T3 seed were harvested for amino acid analyses. The different alleles from each genome are numbered, and the edits identified in each allele are shown in Figure 2. The numbers in parenthesis give the predicted length in amino acid residues of the encoded protein

Line	Plant	A genome alleles	B genome alleles	D genome alleles
23	33	A1 (81)	B1 (39)	D1 (81)
		A2 (97)	B2 (160)	
	60	A1 (81)	B1 (39)	D1 (81)
	75	A1 (81)	B1 (39)	D1 (81)
		A2	B2 (160)	
59	6	A7 (577)	B5 (160)	D4 (76)
		A8 (80)		
	16	A7 (577)	B5 (160)	D4 (76)
	26	A7 (577)	B5 (160)	D4 (76)
	59	A7 (577)	B5 (160)	D4 (76)
		A8 (80)		
	84	A7 (577)	B5 (160)	D4 (76)
		A8 (80)		
178	23	A11 (82)	WT (581)	WT (581)
	35	A11 (82)	WT (581)	WT (581)
	51	A11 (82)	WT (581)	WT (581)

Free amino acids were analysed in wholemeal flour produced by grinding whole T3 grains harvested from the selected plants, the availability of more grain from this generation enabling flour rather than single seeds to be analysed. HPLC was used instead of GC‐MS due to a change in methodology at the analytical laboratory. The whole dataset is given in Data S4 (Sheet 5). The free asparagine concentrations in the flour prepared from the grain of individual plants are shown graphically in Figure 3c and as a percentage of the total free amino acid pool in Figure 3d.

The free asparagine levels in this dataset were higher than for the T2 seed, both *per se* and as a percentage of the total free amino acid pool. For the wildtype plants, free asparagine concentration ranged from 14 to 17 mmol/kg (again rounding to two significant figures) (mean 15 mmol/kg), representing 41% of the free amino acid pool, compared with 8.0% in the T1 generation. The free asparagine concentrations for plants of lines 23, 59 and 178 were also higher than in the T2 seed but remained lower than in the wildtype, ranging from 7.8 to 10 mmol/kg in line 23 (mean 8.6 mmol/kg), 3.7 to 10 mmol/kg in line 59 (mean 7.2 mmol/kg) and 8.0 to 13 mmol/kg in line 178 (mean 10 mmol/kg). The means for lines 23, 59 and 178 represent 57, 48 and 68%, respectively, of the mean for the wildtype plants. Free asparagine represented 29% of the total amino acid pool in line 23, 17% in line 59 and 36% in line 178.

Free asparagine as a proportion of the total free amino acid pool rose compared with the T2 seed, despite the fact that the total free amino acid concentration also increased, ranging from a mean of 28 mmol/kg in the plants of line 178 to 41 mmol/kg in the plants of line 59 (more than double that seen in the T2 seed), with the mean for wildtype at 37 mmol/kg. Free asparagine and total free amino acid levels in plants may increase with stress (Curtis *et al.,* 2014; Lea *et al.,* 2007) and it is possible that the increases observed here were caused by the glasshouse getting hot in the summer of 2019 as the seeds were developing. Regulations covering gene edited plants in the European Union meant that the plants could not be removed from the containment glasshouse to a cooler location. Nevertheless, the free asparagine concentration in the edited lines continued to be much lower than in the wildtype, both *per se* and as a proportion of the free amino acid pool.

Statistical analysis of the data showed no evidence of differences between pots, so a relatively simple Analysis of variance (ANOVA) was applied to examine differences between the lines. This showed the effect of line to be significant (*F pr* = 0.04). For the separate lines, the difference in free asparagine concentrations between both triple null lines, 23 and 59, and wildtype was shown to be significant (*p* = 0.0402 for line 23 and *p* = 0.0122 for line 59, *t*‐test) while that for the A genome null line, 178, was marginally significant (*p* = 0.0919, *t*‐test). Means tables for the lines are given in Data S5 (Sheet 6).

**Figure 4:**
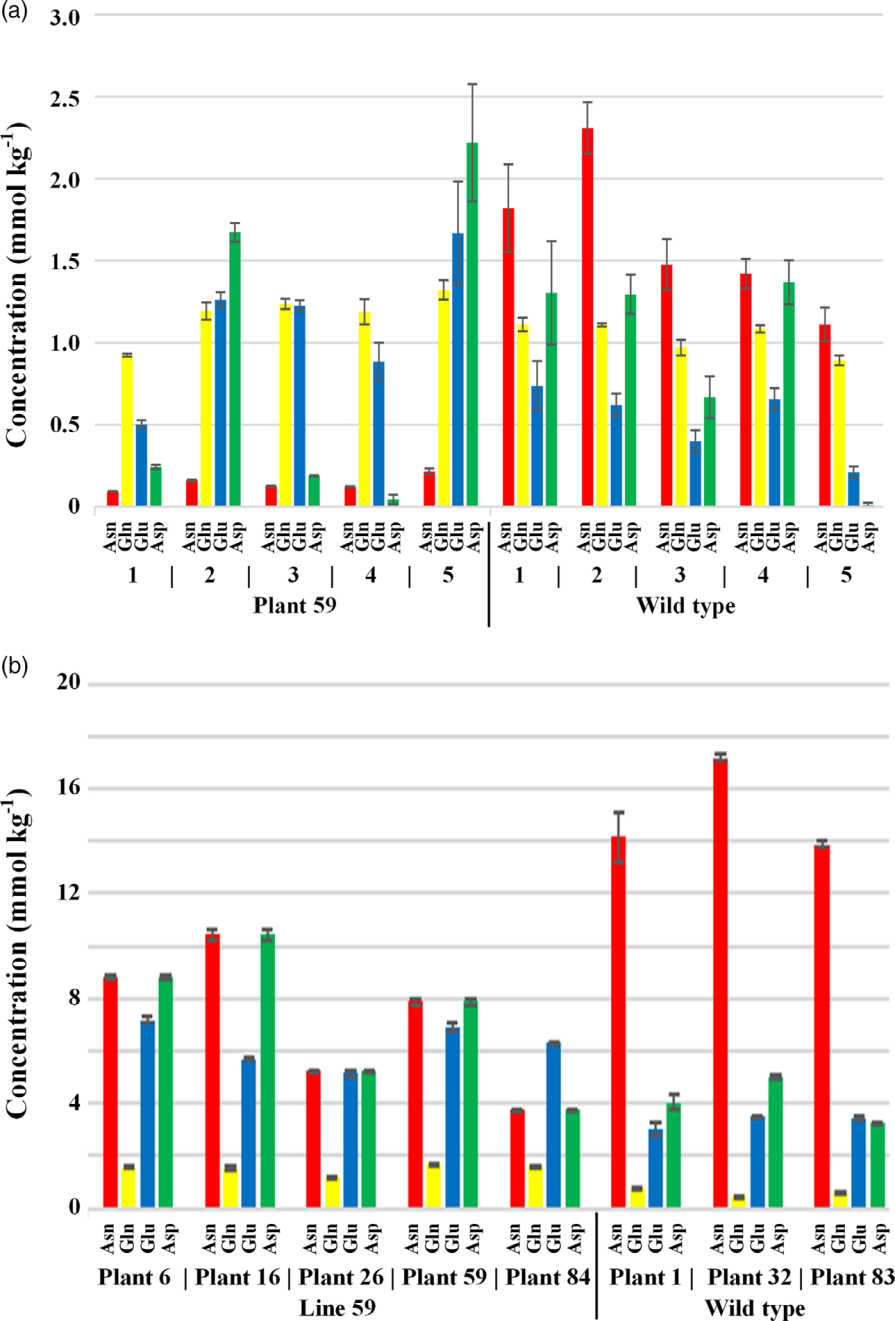
Free asparagine, glutamine, glutamate and aspartate concentrations in the grain of edited wheat (*Triticum aestivum*) cv. Cadenza plant/line 59 and wildtype controls. (a) T2 generation, with free amino acid concentrations shown for five individual seeds from plant 59 and wildlife. (b) T3 generation, with free asparagine concentration shown for wholemeal flour prepared from the grain of five different plants for line 59 (i.e. the progeny of plant 59) and wildtype, as indicated. The graphs show the means and standard errors from three technical reps.

The reaction catalysed by asparagine synthetase involves glutamine and aspartate as the donor and receptor, respectively, of an amino group, and glutamate as the other product in addition to asparagine. The levels of these amino acids are not controlled entirely or even principally by this reaction, of course, but comparing their levels (Data S3 and S4, Sheets 4 and 5) to that of free asparagine in the T2 seeds and T3 flour provided a further striking contrast between the edited lines and the wildtype. Plant 59 showed the sharpest contrast, and the concentrations of free asparagine, glutamine, glutamate and aspartate for the five T2 seeds of this line that were analysed are shown graphically alongside the wildtype in Figure 4a, while the concentration of the same free amino acids in the flour prepared from T3 seeds of five plants of line 59 are shown in Figure 4b.

In the T2 seeds, the ratio of free asparagine to the other free amino acids was dramatically lower in plant 59 than in wildtype (Figure 4a). There was a trend for free glutamate and to a lesser extent glutamine concentration to be higher, while free aspartate concentration was very variable. In the T3 flour, the ratio of free asparagine to the other free amino acids was again much lower for plants of line 59 than the wildtype, although the difference was not so great as in the T2 seeds. The higher concentration of free glutamate and free glutamine in line 59 compared with wildtype was now more pronounced and consistent in the T3 data, and free aspartate was also consistently higher than in wildtype (Figure 4b). ANOVA of the T3 seed data showed the effect of line to be significant for aspartate (*F pr* = 0.007) and glutamate (*F pr* = 0.001), and marginally significant for glutamine (*F pr* = 0.078). For line 59, the differences in free glutamate and aspartate were significant at the 5 % level (means tables and least significant differences (5 %) are given in Data S5, Sheet 6).

### Phenotypic analysis of the T2 plants

The plants in the randomized pot experiment were also assessed for phenotypic effects. An immediate phenotype was noted in that seeds of plants 23, 41 and 59 showed poor germination. This was overcome by an exogenous application of 0.1 M asparagine in water, applied as a spray over the surface of the compost. Subsequently, the plants were assessed for height of tallest tiller, tiller and ear number, above ground biomass, apportioned to stem and ears, and fifty grain weight. The data were analysed using a restricted maximum likelihood (REML)/linear mixed model method. The only variable to show a significant difference (*p* < 0.05, *F*‐test) was the fifty grain weight, which was 2.15 g for line 23, 2.14 g for line 178 and 2.73 g for line 99, compared with 1.68 g for wildtype. At 1.80 g, line 59 was not significantly different (*p* > 0.05) from wildtype for this measure. The full means table is given in Data S6 (Sheet 7).

### *Cas9* segregation in the T2 plants

The presence of the *Cas9* gene in the T2 plants was assessed by PCR to assess whether the *Cas9* gene was segregating away to produce *Cas9*‐free plants. The *Cas9*‐containing plasmid (pRRes.486) that had been used in the wheat transformation was included as a positive control. No product was amplified from DNA of 20 untransformed wildtype plants, while the numbers of *Cas9*‐free plants in the edited lines were two out of ten for line 23, two out of ten for line 41, one out of eleven for line 59 (plant 84, which also had the lowest concentration of free asparagine in the grain; Figure 3c), seven out of nine for line 99 and six out of ten for line 178.

## Discussion

Genome editing with CRISPR/Cas9 is still a relatively novel technique (Jinek *et al.,* 2012) and examples of its successful application in wheat remain few. The use of the technology in wheat protoplasts was described in 2014 (Shan *et al.,* 2014), and Wang *et al*. (2014) used it to knock out the A genome *TaMLO1* gene, which is involved in powdery mildew resistance. Improved powdery mildew resistance was also the aim of a later study that used CRISPR/Cas9 to knock out the *TaEDR1* (*Enhanced disease resistance‐1*) gene, producing five mutant lines, with three edited in all three genomes (Zhang *et al.,* 2017). As in this study, particle bombardment was used to deliver the gRNA construct and, like *TaASN2*, *TaEDR1* is a single copy gene in all three genomes. However, only a single gRNA was used, whereas four gRNAs were used here.

A demonstration of the potential of CRISPR/Cas9 was achieved by Sánchez‐León *et al*. (2018), again using particle bombardment and with two gRNAs delivered separately. They targeted genes encoding α‐gliadins, seed storage proteins with an epitope associated with coeliac disease. Twenty‐one mutant lines were produced in bread wheat and six in durum wheat, all showing a strong reduction in α‐gliadins with up to 35 genes edited in a single line.

*Agrobacterium tumefaciens*‐mediated transformation has also been used to deliver gRNAs into wheat cells, for example to target the *TaPDS* gene, which encodes phytoene desaturase (Howells *et al.,* 2018). Multiple gRNAs were delivered in separate transformations. Interestingly, editing was shown to continue beyond the T0 generation in barley (*Hordeum vulgare*) in that study, but not wheat. Zhang *et al*. (2019) also used *Agrobacterium tumefaciens*‐mediated transformation, and a wheat‐optimized *Cas9* gene, similar to the one used in this study. Sixty‐eight mutants were produced for four grain‐regulatory genes and, in contrast to the Howells *et al*. (2018) study, editing continued into the T1 and T2 generations. One of the genes targeted in that study, *TaGW2*, was also the target of a study by Liang *et al*. (2017) but in that case the gRNAs and Cas9 protein were introduced directly as ribonucleoprotein complexes. A mutation rate of 4.4% was achieved with one host genotype, despite the fact that the technique does not allow for the incorporation of a selection step.

Our study achieved a high mutation rate, with 11 lines derived from the 14 selected T0 plants showing editing. We put this down to the simultaneous introduction of four gRNAs along with the wheat‐optimized *Cas9* gene. Multiplexed gRNAs were also used by Camerlengo *et al*. (2020) to target two genes encoding α‐amylase/trypsin inhibitors in durum wheat, but that study did not involve the use of a selective marker gene, so is not directly comparable. Our study also highlighted some of the problems associated with applying the technique to a hexaploid species, including the size and depth of the NGS data required to characterise the edits, and the reappearance of wildtype alleles in the T2 generation of some lines when none had been detected in the T1 generation. As was reported by Zhang *et al*. (2019), most of the edits were deletions, the longest of which was of 173 base pairs. However, there were some insertions as well, all of a single base pair, and some substitutions. Also as reported by Zhang *et al*. (2019), editing continued beyond the T0 generation, but only in some of the lines. Continued editing increases the chances of effective edits being generated, but also adds to the difficulties of characterising the mutations that have occurred. We conclude that several generations may be required to achieve stability.

Despite these challenges, the study achieved its aim of producing wheat plants with a step reduction in free asparagine concentration in the grain, with concentrations in seeds of T1 plant 59 (i.e. T2 seeds) less than 10% those of wildtype, and concentrations in flour prepared from T3 seeds less than 50% of wildtype. Importantly, some of the plants shown to be low in free asparagine were *Cas9*‐free by the T2 generation. The fact that free asparagine concentration increased in the T3 seed compared with the T2 seed, probably as a result of heat stress, despite the lack of a functional *TaASN2* gene, suggests that one or more of the other asparagine synthetase genes was involved. It is important that the levels in the edited lines continued to be substantially lower than wildtype despite this because it means that the lines are more likely to be consistently low in asparagine under field conditions.

Other stresses that cause an increase in free asparagine concentration include sulphur deficiency (reviewed by Raffan *et al.,* 2020). The sometimes very large increases in free asparagine associated with that stress are accompanied by an increase in total free amino acids and in particular an increase in free glutamine concentration. In contrast, reduced free asparagine concentration in the edited lines in this study was associated with increases not only in free glutamine but also in free glutamate and aspartate. In other words, the partitioning between free asparagine, glutamine, aspartate and glutamate was altered.

The relatively complex nature of the wheat asparagine synthetase gene family lent itself to the aims of the study. *TaASN2* is one of five single copy genes per genome in wheat and is the only one to be expressed seed‐specifically (Curtis *et al.,* 2019; Gao *et al.,* 2016). Non‐Triticeae cereals, such as maize and rice, do not have an equivalent gene (Raffan and Halford, 2021), and rice lines lacking a functional *OsASN1* gene (equivalent to *TaASN4*) showed effects on plant height, root length and tiller number (Luo *et al.,* 2019). There was evidence of increased seed size in some of the edited lines in this study, although not in line 59, which had the lowest free asparagine concentration. Otherwise, the only developmental phenotype was poor germination in some of the low asparagine lines and this could be overcome by application of asparagine. This effect could have implications for pre‐harvest sprouting and malting, but needs to be researched further. A caveat to the observation of a lack of other phenotypic changes is that the low asparagine lines have not been tested in the field yet. Nevertheless, the results of the study give great encouragement that very low asparagine commercial wheat varieties could be developed, facilitating the production of bread, biscuits, breakfast cereals and other wheat‐based foods with lower levels of acrylamide.

## Experimental procedures

**Figure 5:**
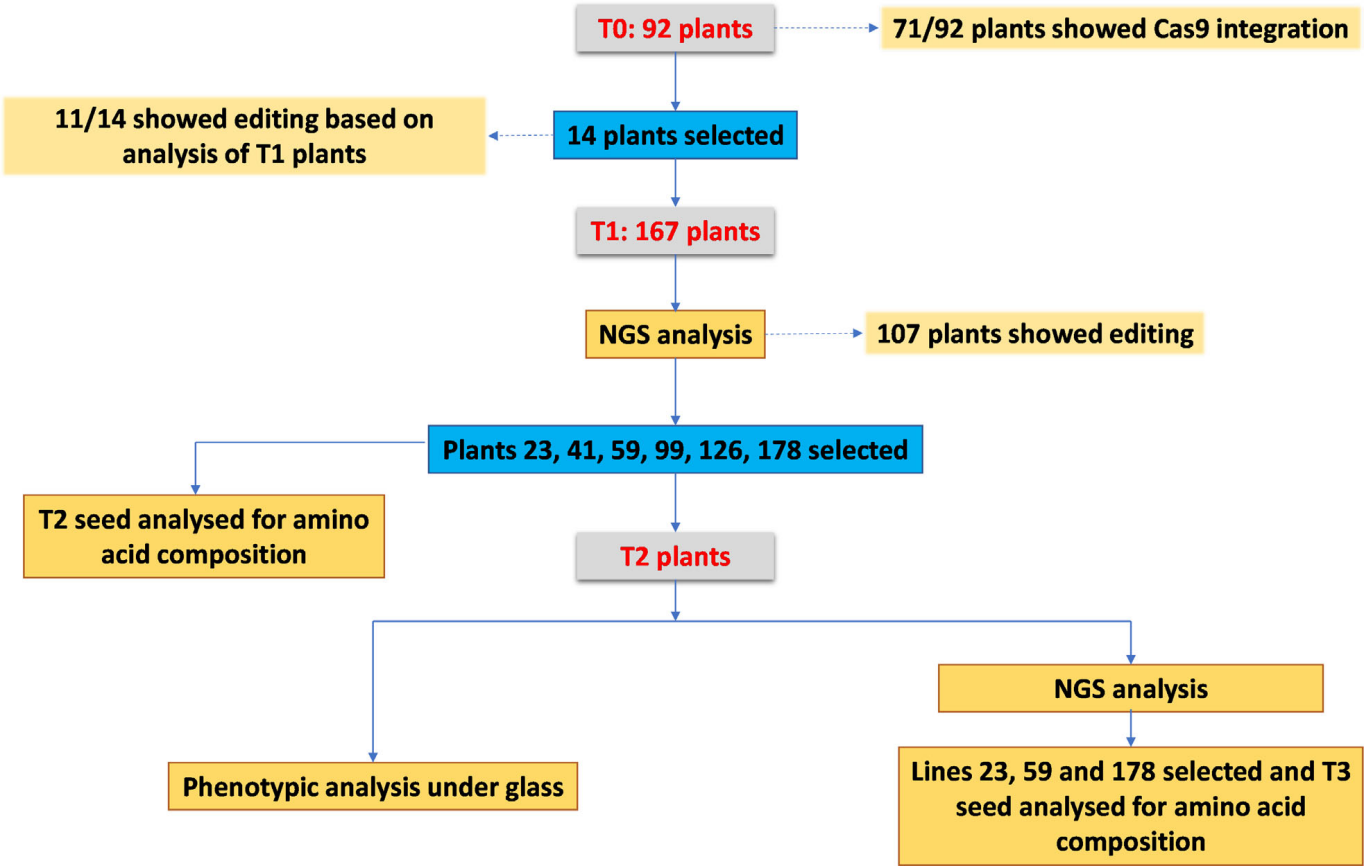
Workflow diagram of the experimentation.

A workflow diagram for the experiments is given in Figure 5.

### Plasmid vectors

DNA fragment purification from agarose gels or PCR reactions was performed using the Wizard^®^ SV Gel and PCR Clean‐Up System (Promega, Southampton, UK). Minipreps were performed using the Wizard^®^ Plus SV Minipreps DNA Purification System (Promega). DNA was quantified using a NanoDrop 2000 spectrophotometer (Thermo Fisher, Hemel Hempstead, UK). Nucleotide sequence analysis was performed by Eurofins Genomics (Wolverhampton, UK). Chemically competent DH5α and 10‐beta cells were acquired from New England Biolabs (Hitchin, UK) and stored at −80 °C before being transformed using a ‘heat shock’ procedure.

Golden Gate (GG) assembly of plasmid constructs was as described by Xie *et al*. (2015). PCR for GG fragment generation was performed in 50 µL reactions using the Q5 High‐Fidelity 2 × Master Mix (New England Biolabs), pGTR gRNA template and 0.5 µM primers (Data S2, Sheet 3), with annealing temperature of 50 °C. The fragments (25 ng) were then used in the GG reaction and the GG product reamplified using the ThermoFisher 2 × ReddyMix PCR Master Mix, primers S5AD5‐F1 and S3AD5‐R1, annealing temperature 60 °C. The product was digested with *Fok*1 (New England Biolabs), then ligated into pRRes209.481.

### Genetic transformation of wheat (*Triticum aestivum*) cv. Cadenza

Transformation of immature embryos was performed by the Cereal Transformation Group, Rothamsted Research, using a PDS‐1000/He™ Biolistic Particle Delivery System (BioRad, Watford, UK), as described (Sparks and Doherty, 2020). Embryos were transferred to selective medium containing glufosinate ammonium after one week and then to fresh selective medium every two to three weeks. Once plants were established they were transferred to soil (Rothamsted prescription mixture), and grown in a containment glasshouse. Glasshouse temperatures were maintained at 22 °C day/18 °C night for 3–4 weeks, followed by 20 °C day/15 °C night for continued growth. Supplementary lighting was used when necessary to provide the plants with a 16‐hour day. Plants were self‐pollinated and grain was collected at maturity.

### DNA extraction from leaves

Nuclei lysis solution (Promega, 600 µL) was added to ground leaf samples and the suspension vortexed, then incubated at 65 °C for 15 min. RNAseA was added (1.5 µL, 10 mg/mL solution) and the reaction mixed, then incubated at 37 °C for 15 min before cooling to room temperature. Protein precipitation solution (Promega, 200 µL) was added and the tubes vortexed for 20 s before centrifugation at 16 000× *g* for 3 min. DNA was precipitated from the supernatant with isopropanol, washed with 70% ethanol, then resuspended in sterile distilled water.

### Next Generation Sequencing (NGS) analyses

The target region of the *TaASN2* gene was amplified from leaf DNA by PCR using primers 5′‐GTAGAGCCAAGCCATTCCTG and 5′‐GCGATGACCTCGCAGTCAC. Adapters (4 bps) were added at the 5′ end to act as barcodes, with a unique barcode for each plant, allowing reads to be assigned. NGS was performed using a MiSeq v2 Benchtop Sequencing System (Illumina, Cambridge, UK) at the Bristol Genomics Facility (University of Bristol) using TruSeq DNA Nano Kits and MiSeq Reagent Kits v2 Nano (Illumina). PANDAseq version 2.11 (Masella *et al.,* 2012) was used to assemble the original fastq paired reads into contigs and demultiplexed by plant using a bespoke perl script. The fasta file for each plant was aligned to the reference sequence using a Bowtie 2 build version 2.3.4.1 (Langmead and Salzberg, 2012).

### Amino acid analyses

Individual seeds from T1 plants (i.e. T2 seeds) were dried, weighed and ground to powder under liquid nitrogen. Pre‐chilled solvent A (500 mL methanol, 200 mL chloroform, 200 mL water) was added (1 mL per 50 mg powdered seed), the solution was vortexed and left on ice for 30 min, vortexed again and centrifuged at 12100 × *g*, 2 min, in a benchtop microfuge. The supernatant was removed and placed on ice while the pellet was re‐extracted with cold Solvent B (500 mL methanol, 500 mL chloroform). The resuspended pellet was vortexed and then left on ice for 30 min, vortexed again and spun at 12100 × *g*, 2 min. The two supernatants were combined and the phase s separated by addition of chilled water. Free amino acids were derivatized using EZfaast Amino Acid Analysis Kit (Phenomenex, Macclesfield, UK) and analysed using an Agilent 6890 GC‐5975‐MS system (Agilent, Santa Clara, CA) in electron impact mode, as described (Curtis *et al.,* 2018b; Elmore *et al.,* 2005). Data were generated using the Agilent Chem Station app.

Whole grains from the T2 plants (T3 grain) were milled to wholemeal flour in a coffee grinder. Free amino acids were extracted as described (Curtis *et al.,* 2018b), derivatized with o‐phthalaldehyde (OPA) and analysed by HPLC, as described by Noctor and Foyer (1998), using an Agilent 1100 HPLC system (Agilent Technologies, US), equipped with a Kinetexcolumn (2.6 µm XB‐C18 100 Å LC Column, 150 × 4.6 mm; Phenomenex, UK) with an FLD detector (Agilent G1321D). Excitation and emission wavelengths were 340 nm and 455 nm, respectively. Eluents were as follows: (A) 80% 50 mm sodium acetate (pH 5.9) in 19% methanol and 1% tetrahydrofuran (THF); (B) 80% methanol in 20% 50 mm sodium acetate (pH 5.9) at 0.8 mL/min flow rate. Run time was 46 min, with the column heated at 30 °C. Run conditions: eluent A 100%, 0–1 min; eluents A (90%) and B (10%), 6–11 min; eluents A (55%) and B (45%), 16–20 min; eluent B (100%), 32–40 min; eluent A (100%), 41–46 min. Data were obtained and analysed using Chemstation32 (v. B.04.03; Agilent Technologies).

### Statistical analyses

Statistical analyses were performed using GenStat (2018, 19^th^ edition, © VSN International Ltd, Hemel Hempstead, UK).

### Diagnostic PCR for the presence of *Cas9*

DNA was isolated from leaf samples and quantified using the NanoDrop^®^ ND‐1000 UV‐Vis Spectrophotometer (Thermo Fisher, Hemel Hempstead, UK). The 2 × ReddyMix PCR Master Mix (Thermo Scientific) was used, with an annealing temperature of 57 °C and 100 ng/µL plant DNA. The primers were UbiPro4 (5′‐TTTAGCCCTGCCTTCATACG) and Cas9‐SR1 (CACCTTCGCCATCTCGTTGC).

## Conflict of interest statement

The authors had complete control of the design of the study, the collection, analysis and interpretation of data, the writing of the manuscript and the decision to publish, without interference from the funders and partner organizations listed in the acknowledgements.

## Author contributions

SR conducted the experiments and analysed the data; CS, AH, LH and DM were involved in gRNA design, plasmid construction and wheat transformation; AM was responsible for statistical analysis of the data; SJH, PAW and GB generated the NGS data on the edited lines; TYC, SU and OK produced the data on free amino acid concentrations in the grain; KJE and NGH were the project leaders and were responsible for the conception and design of the study; SR and NGH drafted the manuscript.

## Supporting information


**Data S1**. Ensembl Plant database (http://plants.ensembl.org/index.html) references for the asparagine synthetase genes of wheat (*Triticum aestivum*) cv. Cadenza (Sheet 2).
**Data S2**. Primers used for Golden Gate (GG) assembly (Sheet 3).
**Data S3**. T2 seed free amino acid concentrations (Sheet 4).
**Data S4**. T3 seed free amino acid concentrations (Sheet 5).
**Data S5**. Results from analysis of variance (ANOVA) of the T3 seed data for free asparagine, aspartic acid, glutamic acid and glutamine (Sheet 6).
**Data S6**. Table of means from restricted maximum likelihood (REML) analysis of phenotype data (Sheet 7).
